# The height of the operating table affects the performance of residents in combined spinal and epidural anesthesia training by affecting the vision of the puncture needle: a randomized controlled trial

**DOI:** 10.1186/s12871-023-01985-6

**Published:** 2023-01-17

**Authors:** Juan Gu, Juan Ni, Yushan Ma, Yaqin Xiong, Jin Zhou

**Affiliations:** Department of Anesthesiology, West China Second University Hospital, Sichuan University; Key Laboratory of Birth Defects and Related Diseases of Women and Children (Sichuan University), Ministry of Education. 20#, Section 3 Renmin Nan Road, Chengdu, Sicuan, Chengdu, 610041 China

**Keywords:** Residents training, Combined spinal-epidural anesthesia, Table height

## Abstract

**Background:**

The present study aimed to evaluate whether the operating table height affected the success rate and incidences of complications of combined spinal-epidural anesthesia administered by residents during training.

**Methods:**

One-hundred-and-eighty patients were randomly allocated according to landmarks on the resident’s body: umbilicus (group U), lowest rib margin (R), and xiphoid process (X). The success rates of combined spinal-epidural anesthesia, and the incidences of paresthesia and vessel trauma were recorded.

**Results:**

There were no differences between the three groups in the success rates of combined spinal-epidural anesthesia, and the incidences of paresthesia and vessel trauma. However, paresthesia during epidural catheter advancement was more common on the left side (66.7%) than the right side (33.3%) (*P* = 0.03). In group R, the success rate of epidural anesthesia was higher during the residents’ third time (100%) than their first time (50%; *P* = 0.01). Most residents (83%) preferred the table height at which the needle insertion point was at the level of their lowest rib margin.

**Conclusions:**

Neither the success nor the complication of combined spinal-epidural anesthesia in lateral decubitus position during residents’ training affected by the operating table height. However, paresthesia was more likely to occur on the left side when a stiff catheter was inserted into the epidural space. It may be better to keep the table height at residents’ lowest rib margin. It was not just preferred by most of residents but also better for their training of performing epidural anesthesia.

**Trial registration:**

The trial was registered prior to patient enrollment at Chinese Clinical Trial Registry (NCT: ChiCTR1800016078, Principal investigator: Juan Gu, Date of registration: 9 May 2018). Registry URL http://www.chictr.org.cn

## Background

Combined spinal-epidural anesthesia (CSEA) combines the advantages and mitigates the disadvantages of single-shot spinal anesthesia and continuous epidural anesthesia, and is an essential component of neuraxial anesthesia in anesthesiology residency training. The most popular CSEA technique is the needle-through-needle technique in which the epidural needle serves as an introducer for the spinal needle. Puncture in lateral decubitus position is a common method of CSEA.

The height of the operating table affects the comfort and performance of the operator [1, 2], and the angle formed between the spinal needle and skin on the patient’s back in the coronal plane [3]. The table height is more likely to affect the spinal needle in the needle-through-needle technique in CSEA compared with spinal anesthesia in lateral decubitus position, as the success of spinal anesthesia depends upon the angle of the epidural needle in the epidural space [4].

Furthermore, the vision interference induced by table height may affect residents more than experienced clinicians. It remains unclear whether this vulnerability would decrease after a period of training.

The purpose of the present study was to evaluate whether the operating table height affected the success rate and incidence of complications for CSEA in lateral decubitus position performed by residents during training.

## Methods

The present study was conducted with the approval of the China Ethics Committee of Registering Clinical Trials (ChiECRCT-20,180,065), and written informed consent was obtained from all subjects participating in the trial. The trial was registered prior to patient enrollment at Chinese Clinical Trial Registry (NCT: ChiCTR1800016078, Principal investigator: Juan Gu, Date of registration: 09 /05/2018). This study also adheres to the CONSORT guidelines.

100-and-8 pregnant women, ASA I-II, undergoing CSEA for cesarean delivery were recruited and provided written informed consent. The exclusion criteria were contraindications for CSEA, and history of spinal surgery or severe anatomical abnormalities of the spine. Computer-generated block randomization was used to randomly allocate the patients to one of three groups in accordance with the height of the operating table. The table height was set with the needle insertion point (the midline of the patient’s body) at the level of the anesthesia provider’s umbilicus for group U, the lowest rib margin for group R, and the xiphoid process in the standing posture for group X.

Anesthesia was administered by residents who had each performed neuraxial anesthesia less than 10 times. Exclusion criteria for residents were: body mass index > 30, pregnancy, musculoskeletal disorders, and refusal to participate.

Residents completed a 2-month training period for CSEA. After theoretical learning and simulated training, each resident was scheduled to practice CSEA in the obstetric surgery room for 2 days each week. Before initiating CSEA, an anesthesiologist who was not blinded to group allocation adjusted the height of the operating table in accordance with the grouping information contained in sealed opaque envelopes. Patients were placed in the left lateral decubitus position, parallel to the edge of the operating table, with the knees flexed on the abdomen, and the neck flexed. Under supervision of an anesthesiologist, the resident inserted a 17-gauge Quincke epidural needle (YA GUANG, China) orientating patients’ head in the midline at the L3–L4 or L2–L3 interspace. The epidural space was identified using the loss-of-resistance method. After the epidural needle was positioned in the epidural space, a 25-gauge spinal needle was advanced through the epidural needle, dural puncture was verified by visualization of cerebrospinal fluid after removal of the spinal needle stylet, and 10–12.5 mg bupivacaine was administered. A stiff catheter was inserted into the epidural space after the spinal needle was removed. Epidural vein cannulation was verified by the visualization of blood. If the resident could not successfully advance the needle into the epidural space after two attempts, the epidural anesthesia was classified as a failure and the CSEA was administered by the supervising anesthesiologist. If the resident could not successfully insert the spinal needle or the patient reported the onset of paresthesia while the spinal needle was being advanced, the resident could change the direction of spinal needle advancement twice by depressing or lifting the tail of the epidural needle. If the patient still reported abnormal feelings or the spinal needle still could not be successfully inserted, the spinal anesthesia was classified as a failure and the CSEA was administered by the supervising anesthesiologist.

The primary outcomes were the rates of successful epidural anesthesia and spinal anesthesia. Complications including paresthesia, which was defined as an abnormal feeling (electrical sensation, numbness, and/or pain) in the patient’s lower extremities while the spinal needle was being advanced, and catheterization of a blood vessel were second outcomes. Other secondary outcomes were the time between puncture initiation and the achievement of successful epidural anesthesia, incidence of needle angle adjustment, and the residents’ table height preferences.

A pilot study (10 patients each in groups U and X) was conducted to enable the estimation of an appropriate sample size. The rate of successful CSEA was about 54 and 40%. To detect a difference of 10% in the rate of successful dural puncture after needle entry into the epidural space, 50 patients per group were required to achieve a power level of 80% using a two-tailed test with α = .05. Assuming a dropout rate of 10%, a total of 180 patients were needed to detect a significant difference.

### Statistical analysis

Statistical analyses were performed using one-way analysis of variance, the Mann-Whitney U test, the χ^2^ test, Fisher’s exact test, and logistic regression analysis. *P* < .05 was considered statistically significant. Statistical analyses were performed using SPSS 17.0 for Windows.

## Results

### Demographic data of the patients and residents

**Fig. 1:**
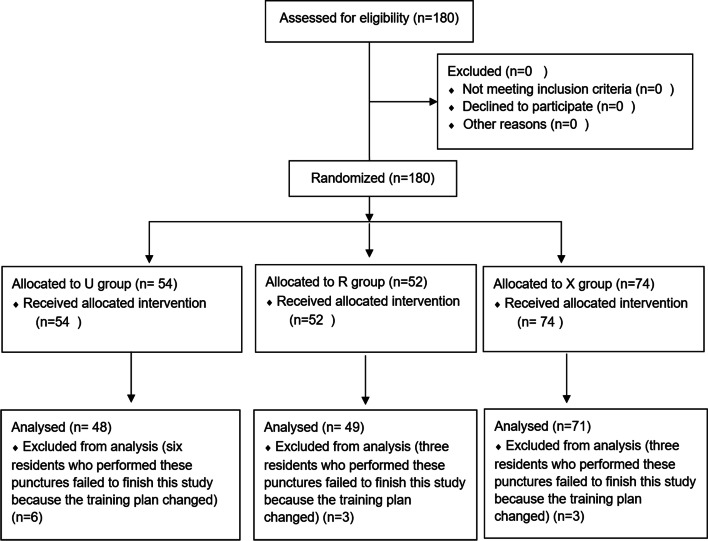
Flow Diagram of this study

One-hundred-and-eighty patients and 25 residents were recruited; however, six patients in group U, three in group R, and three in group X were excluded because the six residents who performed these punctures failed to finish this study because the training plan changed (see Fig. 1). The demographic data of the patients and residents did not differ between the three groups (Tables 1, 2).

**Table 1:** Characteristics of pregnant women in whom combined spinal-epidural anesthesia was administered at different operating table heights

	U (***n*** = 48)	R (***n*** = 49)	X (***n*** = 71)
**Age (yr)**	32.6 ± 5.4	32.7 ± 4.1	32.9 ± 5.0
**Gestational weeks**	38.2 ± 1.3	38.5 ± 1.3	38.3 ± 1.3
**BMI (kgm-2)**	26.5 ± 2.7	26.3 ± 2.8	26.9 ± 2.7

Values are expressed as mean, *BMI* body mass index

**Table 2:** Characteristics of the residents who administered combined spinal-epidural anesthesia

	U (***n*** = 48)	R (***n*** = 49)	X (***n*** = 71)
**Age (yr)**	25.1 ± 1.7	25.1 ± 1.6	24.9 ± 1.7
**Gender (male/female)**	5/43	8/41	18/53
**Height (cm)**	158.5 ± 5.2	159.5 ± 5.3	160.5 ± 6.0
**BMI (kgm-2)**	20.7 ± 1.8	20.6 ± 1.7	20.6 ± 1.6
**Grade (1/2/3)**	18/11/19	17/10/22	25/24/22
**Myopia (y/n)**	29/19	30/19	46/25

Values are expressed as mean or number of subjects, *BMI* body mass index

There were no differences in the success rates of CSEA, epidural space puncture, and puncturing dura after successful epidural needle was sited in epidural space of groups U, R, and X. The times taken to accomplish CSEA and epidural anesthesia did not significantly differ between the three groups. The incidence of paresthesia during advancements of the spinal needle and epidural catheter were similar in all three groups. However, paresthesia more commonly occurred on the left side (66.7%) than the right side (33.3%) during the advancement of the epidural catheter (*P* = 0.03). The incidence of blood vessel catheterization was similar in all three groups. During the process of spinal needle advancement, the incidence of spinal needle directional change (by depressing or lifting the tail of the epidural needle) did not significantly differ between groups U, R, and X. The success rate of dural puncture caused by spinal needle directional change in group R was 100%, which tended to be higher than the success rates in groups U and X, but this difference did not reach statistical significance (Table 3).

**Table 3:** Residents’ performances in administering CSEA at different table heights. Values are expressed as mean or number of subjects

	U (***n*** = 48)	R (***n*** = 49)	X (***n*** = 71)	P
**Success rate of CSEA,** ***n*** (%)	26 (54.2.0%)	29 (58.0%)	34 (47.9%)	0.53
**Success rate of EA,** ***n*** (%)	31 (64.6%)	36 (74.0%)	47 (66.2%)	0.54
**Success rate of SA after**	26 (83.9%)	29 (78.4%)	34 (72.3%)	0.48
**EA success,** ***n*** (%)				
**Times used for CSEA,** sec	359 ± 170	360 ± 203	309 ± 148	0.42
**Times used for EA,** sec	191 ± 144	202 ± 131	154 ± 93	0.17
**Paresthesia rate**				
**Advancing spinal** **needle,** ***n*** (%)	6 (21.4%)	7 (24.1%)	9 (25.0%)	0.94
**Left/ right,** n	4/2	3/4	4/5	1
**Advancing epidural catheter,** ***n*** (%)	5 (19.2%)	7 (25.0%)	8 (23.5%)	0.87
**Left/ right,** n	3/2	5/2	6/2	0.03^#^
**Blood vessel catheteration,** ***n*** (%)	3 (11.5%)	4 (14.8%)	3 (9.4%)	0.91
**Adjustment of spinal needle rate,** ***n*** (%)	7 (22.6%)	8(21.6%)	14 (29.8%)	0.64
**Success rate,** ***n*** (%)	4 (57.1%)	8 (100.0%)	11 (78.6%)	0.09

Success rate of EA, successful epidural space puncture; Success rate of SA, success rate of dural puncture after successful epidural space puncture; Adjustment of spinal needle, change in the direction of the spinal needle advancement by depressing or lifting the tail of the epidural needle. ^#^, paresthesia was more common on the left side than the right

**Fig. 2:**
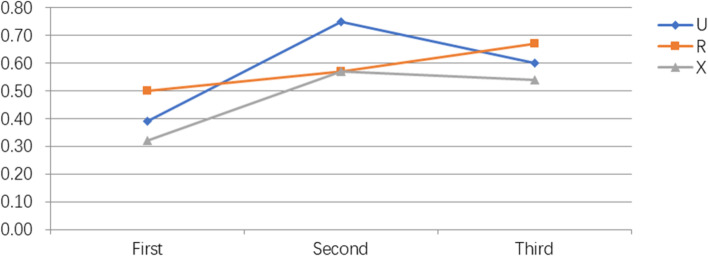
Learning curves of residents performing combined epidural-spinal anesthesia

**Fig. 3:**
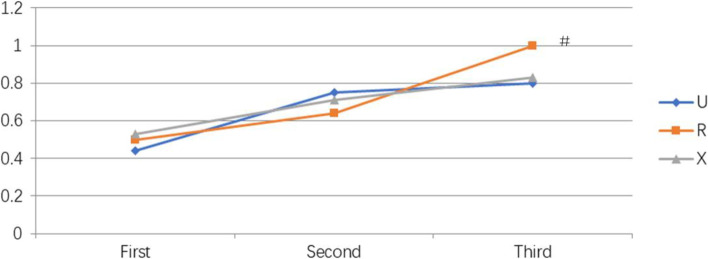
Learning curves of residents performing epidural anesthesia Values are expressed as rates, #, in group R, the success rate of epidural anesthesia administered by a resident during their third time at performing combined epidural-spinal anesthesia was higher than that during their first time (*p* = 0.01)

During resident training for CSEA administration, the success rates of CSEA for the first, second and third time that the residents had performed CSEA were similar in groups U, R, and X. The success rates of CSEA administered by a resident performing CSEA for the third time in each group tended to be higher than that for the first time, but this difference did not reach statistical significance (Fig. 2). The success rates of epidural space puncture administered by a resident during their respective first, second, and third times at performing CSEA were similar in groups U, R, and X. However, in group R, the success rate of epidural space puncture administered by a resident during their third time (100%) was higher than that during their first time (50%; *p* = 0.01; Fig. 3).

### Factors influencing the administration of CSEA

There was no relationship between the success rates of CSEA of each resident and the residents’ age, height, BMI, sex, years of experience, and myopia. Similarly, these resident factors did not affect the incidence of paresthesia.

### Residents’ table height preference

The height of the operating table at which the needle insertion point was set at the level of the operator’s lowest rib margin was preferred by 83% of the residents. This table height was considered more comfortable during the administration of CSEA.

## Discussion

The present study demonstrated that the table height (at the umbilicus, lowest rib margin, and xiphoid process level of the residents administering CSEA) did not influence the success rates of CSEA and epidural space puncture. The table height also did not influence the incidence of complications of CSEA, including paresthesia and blood intravascular cannulation. Paresthesia was more likely to occur on the left side than the right side when the catheter was inserted into the epidural space. The table height at which the needle insertion point was level with the operator’s lowest rib margin was preferred by most residents; this height was also best during training to administer epidural space puncture.

In our study, the success rate of epidural space puncture administered by residents was 68.3%, consist with Drake et al. reported a success rate of 63–90% [5]. In our study, the success rate of CSEA administered by residents was 53.4%, and the success rate of dural puncture after successfull epidural space puncture was 78.2%. One reason for failure of CSEA is that the epidural needle may be angled away from the midline, and so the spinal needle passes to the side of the dural sac [4]. A smaller height difference between the puncture points and the anesthesiologist’s eyes may lead to better visualization, as the line from the anesthesiologist’s eyes to the puncture site is more horizontal. Therefore, as the angle formed between the epidural needle and the skin on the patient’s back in the coronal plane is reportedly greater in groups U and R than in group X [3], this may increase the risk of the epidural needle being angled away from the midline, causing the spinal needle to pass to the side of the dural sac. However, there was no significant difference in the success rate of CSEA between groups U, R, and X in our study. This may be because the difference between the angles formed between the epidural needle and the skin in the coronal plane in groups U, R, and X was too small to influence whether the spinal needle punctured the dural sac. Another potential reason for this lack of significance is that the residents were allowed to regulate the direction of the spinal needles by adjusting the angle between the epidural needle and the patient’s back. However, the incidence of directional change did not significantly differ between the three groups, and the success rates of dural puncture did not significantly differ in accordance with the angle between the epidural needle and the patient’s back.

**Fig. 4:**
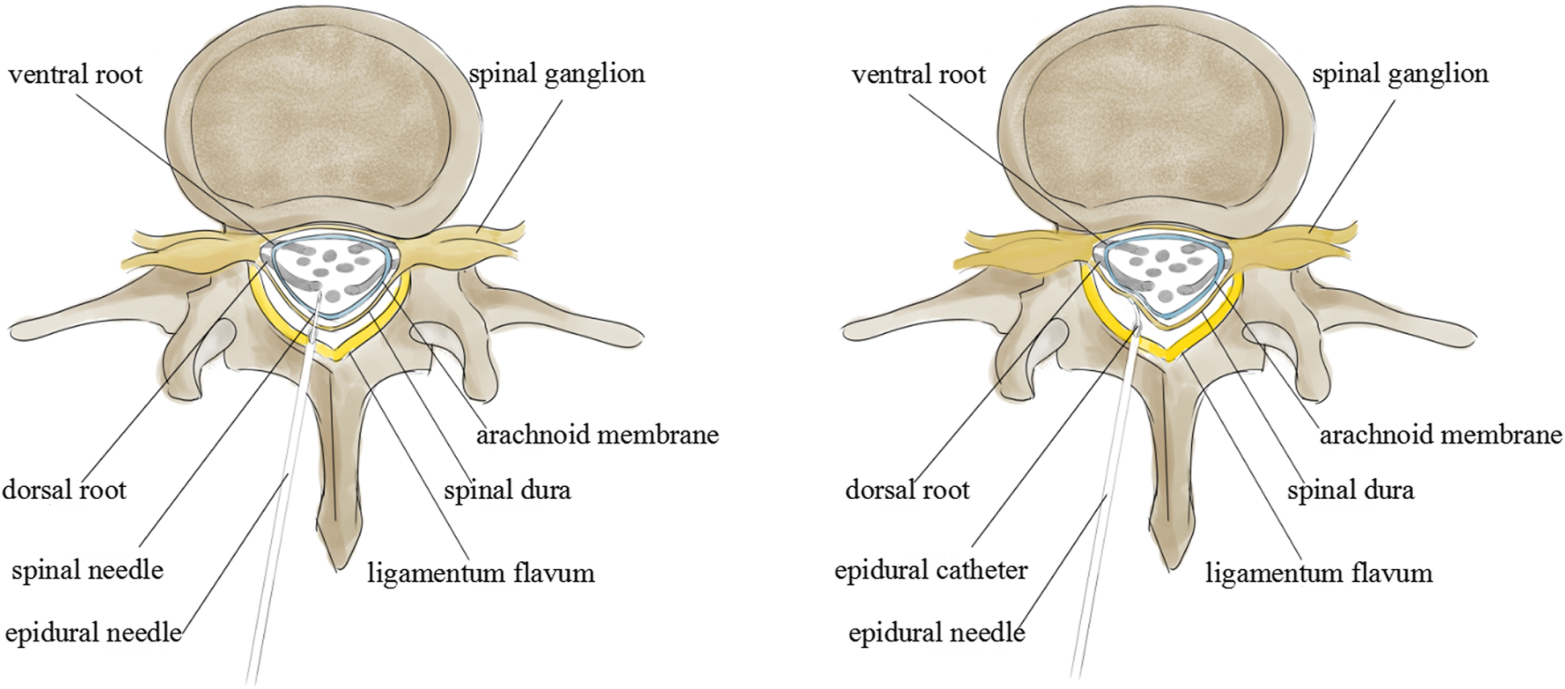
Diagrammatic sketch of combined epidural-spinal anesthesia administrated by most residents

Paresthesia was more likely to appear on the left side than the right side when the catheter was inserted into the epidural space, but there was no difference between sides in the incidence of paresthesia during the advancement of the spinal needle. This may be because the residents were more likely to insert the epidural needle near the left side of the patient, and so the nerve roots on the left side of the epidural space were more likely to be irritated and cause paresthesia when the catheter was inserted. The catheters used in our study were stiff, which may have induced greater incidence of paresthesia and intravascular cannulation than wire-embedded catheters [6–8]. A previous study reported that the angle formed between the patient’s back skin and the needle in the coronal plane was greater than 90° in groups U and R [3]; this might increase the risk of the spinal needle being inserted closer to the right side compared with the epidural needle. Therefore, there was no difference in the incidence of paresthesia on the left versus the right side during the process of spinal needle advancement. (Fig. 4).

There was no relationship between the success rates of CSEA and the residents’ age, height, BMI, sex, experience level, and myopia. Similarly, these factors had no influence on the incidence of paresthesia. This suggests that the demographic characteristics of the residents did not influence their ability to administer CSEA during training; the effectiveness of their training was only influenced by their degree of comfort during the process of performing epidural anesthesia. In contrast, a previous study showed that the resident’s experience level significantly affected the incidence of paresthesia during the advancement of a thoracic epidural catheter [7]. However, this previous study divided the residents into those with less than 4 years of experience and those with more than 4 years of experience, while all of the residents in our study had less than 3 years of experience.

The table height most favored by residents was that at which the puncture point was at the same height as their lower rib margin, as this height was considered more comfortable and thus favorable for the performance of successful CSEA. A working surface height that ranges from 10 cm below to 5 cm above the elbow is optimal for standing workers [9, 10]. The table height at which the puncture point was level with the residents’ lower rib margins were within this range. At this table height, the residents’ upper arms could rest on their chest walls, and the forearms were more stable and less tired when performing CSEA. A previous study reported that the anesthesiologists preferred higher table heights than the residents [3]. Compared with the anesthesiologists, the residents were more vulnerable to upper arm tiredness because of the longer duration of the procedure [3].

The present study had some limitations. First, the number of CSEA procedures performed varied between residents. This was because each resident was assigned to a different surgery room, and the number of CSEA procedures performed by each resident was dependent on the surgeries scheduled on that day. At least 20–25 epidural blocks are reportedly necessary to achieve consistency during residency training in anesthesiology [11]. However, although each resident in our study administered epidural anesthesia less than 20 times during training, we focused on the training process rather than the training results. Second, same resident was not exposed to all three position of needle insertion. Resident often cannot differentiate and tell the best position for performing a procedure due to their experience being limited. Third, the present study did not evaluate the administration of CSEA while the patients were in a sitting position. Fourth, the randomization of residents were not performed, this may be an operator bias. In our hospital, residents are assigned to fixed operating rooms for CSEA training every day. They are trained not only in the operation of CSEA, but also in the management of intraoperative patients. If randomization of residents are performed, they have to switch to other operating rooms, which is not only bad for training but also bad for patient safety. Therefore, we did not randomize the residents. Finally, we did not evaluate the administration of CSEA while the operating table height was above the xiphoid process of the residents. This was because some patients were afraid on operating tables with a height elevated for tall residents.

## Conclusions

In conclusion, neither the success nor the complication of CSEA in lateral decubitus position during residents’ training affected by the operating table height. However, paresthesia was more likely to occur on the left side when a stiff catheter was inserted into the epidural space with the table height is not above the residents’ xiphoid process. It may be helpful to suggest the residents to adjust the angle between the patients’ back skin and the needle to 90°by lowering their bodies to reduce the disturb of vision. It may be better to keep the table height at which the needle insertion point was level with the residents’ lowest rib margin. It was not just preferred by most of residents but also better for their training of performing epidural anesthesia.

## Data Availability

The datasets generated and/or analyzed during the current study are not publicly available before publication of our study, but are available from the corresponding author on reasonable request.

## Abbreviations

CSEA: combined spinal-epidural anesthesia
U: umbilicus
R: rib margin
X: xiphoid
BMI: body mass index
